# Editorial: Multi-omics strategies for salinity and drought stress mitigation in agriculture

**DOI:** 10.3389/fpls.2026.1857467

**Published:** 2026-05-05

**Authors:** Muhammad Waseem, Rafaqat Ali Gill

**Affiliations:** 1 School of Breeding and Multiplication (Sanya Institute of Breeding and Multiplication), School of Tropical Agriculture and Forestry, Hainan University, Sanya, China; 2 Jiangxi Provincial Key Laboratory of Plant Germplasm Innovation and Genetic Improvement, Lushan Botanical Garden, Chinese Academy of Sciences, Jiujiang, China; 3 Plant Epigenetics and Development, Lushan Botanical Garden, Chinese Academy of Sciences, Nanchang, China; 4 College of Life Science, Nanchang University, Nanchang, China

**Keywords:** adaptation, mechanism, multiomic, osmotic adjustment, sustainability

Salinity and drought are among the most detrimental abiotic stressors affecting global crop yields, posing significant threats to food security in the world’s most vulnerable regions. The escalation in the frequency and severity of these stressors, driven by climate change, soil degradation, and intensified land use, highlights the urgent necessity for developing resilient, high-yielding crop cultivars (Waseem et al., 2023; Sahu et al., 2025). In this context, multi-omics technologies have emerged as transformative tools for dissecting the complex molecular networks that underpin plant stress perception, signaling, and adaptation.

Over the past decade, genomics, transcriptomics, proteomics, metabolomics, ionomics, and phenomics have each contributed essential pieces of the salinity and drought tolerance puzzle (Waseem et al., 2025). The integration of these disciplines into a unified multi-omics approach represents a significant advancement, facilitating a systems-level understanding of stress responses that spans from alleles and regulatory modules to pathways, tissues, and entire plant phenotypes (Waseem et al., 2022). This Research Topic, “Multi-Omics Strategies for Salinity and Drought Stress Mitigation in Agriculture”, serves as a critical reference point and inspiration for researchers aiming to harness integrative omics for climate-resilient agriculture. The six papers presented in this Research Topic provide high-resolution insights across diverse plant species, revealing proline metabolism, ion homeostasis (e.g., HKT/SOS/NHX), transcriptional regulation (e.g., CPP, WRKY, NAC), and metabolic reprogramming as conserved tolerance pillars. By employing strategies ranging from Genome-Wide Association Studies (GWAS) and proteomics to immediate agronomic solutions like grafting, these works define precise molecular targets for improving both major and underrepresented crops, including safflower, kiwifruit, okra, tomato, and *Medicago*. Finaly, the integration of these molecular insights with CRISPR editing and AI-driven modeling will accelerate the transition towards a more sustainable and stress-resilient agricultural future. We extend our sincere gratitude to all authors and reviewers for their contributions to this dynamic and rapidly evolving field.

## 1 Genomic and transcriptional insights into stress tolerance

Ali et al. conducted a GWAS on 94 safflower (*Carthamus tinctorius*) accessions from 26 countries to explore the genetics of salt tolerance at seedling stage. They quantified multiple morphological and physiological traits related to salt injury and growth reduction and the GWAS detected 322 significant marker-trait associations across key traits such as biomass, root and shoot weights, leaf number, plant height, and root length. Gene annotation highlighted multiple candidate genes, including *PLA1, APK4, GINT1, TPLATE, SPS2, DTX50*, and *RAF1*, which are involved in secondary metabolite sulfation, chloroplast development, cell wall modification, sucrose biosynthesis, and calcium signaling. In *silico* expression analysis under salinity showed that several of these genes strongly upregulated (such as *PLA1*, *SPS2*, and *DTX50*) or downregulated, supporting their role in salt tolerance. Overall, the study delivered elite salt-tolerant genotypes (G-50, G-58, and G-94) and a set of biologically plausible candidate genes that merit functional validation for safflower improvement.

Zheng et al. systematically identified 65 *CPP* transcription factor (TF) genes across six Theaceae species and grouped them into two subfamilies characterized by conserved CXC domains. The study showed that Class II genes are more structurally conserved, typically with 8 exons, while many Class I genes contained 10 exons, indicating evolutionary divergence. Whole-genome duplication was the primary driver of this family’s expansion, and the gene promoters were found to be rich in stress-related regulatory elements. Expression and qRT-PCR data revealed that specific Class I genes (such as *CsinCPP2*, *CcheCPP1*, and *ColeCPP12*) were highly expressed in vegetative tissues like leaves, apical buds, and stems. Conversely, several Class II genes (including *ColeCPP1*, *CsinCPP9*, *ColeCPP2*, and *CcheCPP8*) were strongly induced by drought and salt stresses, highlighting them as key candidates for growth regulation and stress tolerance. Overall, the work clarifies evolutionary patterns and provided candidate *CPP* genes for functional studies and stress-resilience improvement in Theaceae.

## 2 Transcriptional and proteomic mechanisms of resilience

Yang et al. characterizes key proline metabolism genes in kiwifruit (*Actinidia chinensis*) and linked them to salt stress tolerance. The study identified two *AcP5CS*, one *AcP5CR*, one *AcOAT*, three *AcPDH*, and one *AcP5CDH* gene, and analyzed their evolution, structure, regulatory elements, chromosomal location, and expression under abiotic and hormonal treatments. Under salt stress, *AcP5CS1, AcP5CR*, and *AcOAT* were strongly upregulated, while *AcP5CDH* was repressed, a pattern confirmed by qRT-PCR. Overexpressing *AcP5CS1* in *Arabidopsis* significantly improved salt tolerance, demonstrating its functional importance. The TF *AcNAC30* was found to be highly correlated with *AcP5CS1* expression and directly bound its promoter, indicating that *AcNAC30* likely enhances proline accumulation under salt stress by activating proline biosynthesis genes. Overall, the work defines the proline metabolic gene network in kiwifruit and demonstrates its central role in abiotic stress resilience.

Grafting is a widely used horticultural technique that joins the shoot of one plant (scion) to the root system of another (rootstock), often improving stress resilience. Mahapatra et al. showed that grafting drought-susceptible tomato (*Solanum lysopersicum*) scions onto drought-resistant rootstocks significantly improved drought tolerance. In the initial screening, two resistant genotypes use as rootstocks and susceptible genotypes as scion produced grafts (G1 and G4) that maintained higher relative water content, better chlorophyll fluorescence, and higher stomatal conductance under drought. Physiologically, these successful grafts alleviated drought damage by preserving photosynthetic pigments and reducing oxidative stress. Proteomic analysis indicated enhanced cellular stress responses, metabolism, and defense pathways in the grafted plants. Transcriptomic profiling revealed the upregulated expression of key stress-related genes, including *DREB*, *WRKY*, *PIPs*, *SOD*, *CAT*, *APX*, *HSPs*, and *LOX* in G1 and G4, explaining their superior performance. Overall, the work demonstrates that appropriate rootstock-scion combinations can improve molecular and physiological drought defenses in tomato plants.

## 3 Physiological thresholds and metabolic reprogramming

Yang et al. found that okra (*Abelmoschus esculentus*) germination was highly sensitive to salinity, being fully inhibited at concentrations ≥ 0.5% NaCl, while seedling growth followed a hormetic response, where growth was promoted at mild levels (0.1–0.3%) but suppressed at higher ones. Under moderate salt stress, okra maintained photosynthetic integrity and photoprotection, while severe stress increased oxidative damage. Transcriptome profiling under mild salinity showed the coordinated upregulation of ion homeostasis genes, calcium signaling, and *GH3* auxin-responsive genes (log2FC 2.3–2.5), alongside ROS detoxification, cytoskeletal remodeling, and metabolic shifts to stabilize cells. Overall, the study defined okra’s salinity thresholds and identified molecular targets, such as auxin conjugation pathways, for breeding salt-tolerant varieties suited to marginal saline lands.

Wei et al. examined the seed germination and metabolic responses of four *Medicago ruthenica* germplasm lines (YSZ, XHZ, Shoulu, and Longzhong 1) under NaCl, *Na_2_SO_4_*, *NaHCO_3_*, and mixed saline-alkali stress. The authors found that while low NaCl concentrations promoted germination, high NaHCO_3_ and compound stress strongly inhibited both seed germination and seedling growth. Germplasm tolerance varied by origin, with domesticated lines (specifically XHZ) showing superior salt-alkali resistance. Untargeted LC-MS metabolomics revealed stress-induced shifts in amino acid, sugar, and lipid metabolism, marked by elevated levels of amino acids and their derivatives. Notably, L-arginine, histidine, and glutamine strongly correlated with higher germination rates and root length, indicating their key roles in stress adaptation. Tolerant lines accumulated betaines (3-fold increase), flavonoids/phenolics (2-fold increase), and amino acids, with pathway enrichment in osmoprotection, phenylpropanoid biosynthesis, and ROS scavenging. This metabolic reprogramming sustained significantly (40%) higher germination vigor while reducing oxidative damage, highlighting key biomarkers for saline-alkali forage tolerance screening.

**Figure 1:**
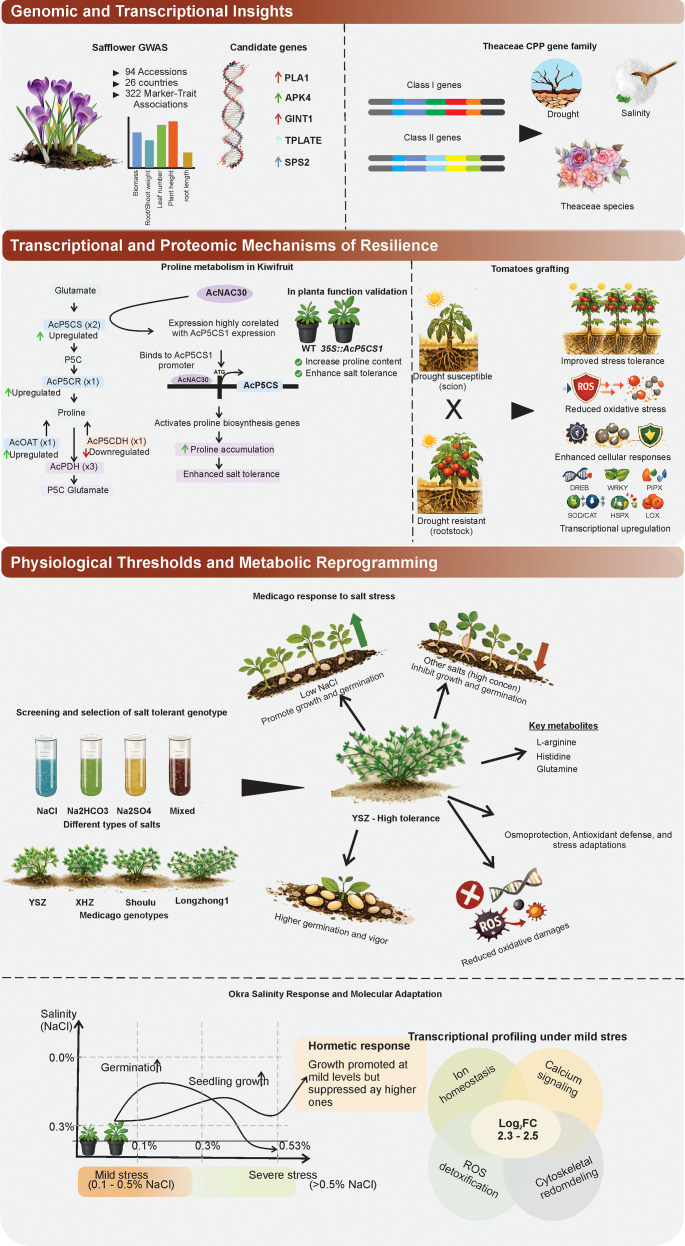
Integrated overview of genomic, transcriptional, proteomic, physiological, and metabolic mechanisms underlying plant stress tolerance. The figure summarizes recent studies on plant responses to salinity and drought stress across multiple species. In the genomic and transcriptional insights section, safflower GWAS identified significant marker–trait associations and candidate genes linked to salt tolerance, while analysis of the Theaceae CPP gene family highlighted stress-responsive class I and class II genes associated with drought and salinity adaptation. In the transcriptional and proteomic mechanisms of resilience section, the kiwifruit proline metabolic pathway is shown with salt-induced upregulation of biosynthetic genes and regulation by AcNAC30, alongside functional validation of enhanced salt tolerance. The tomato grafting model illustrates how drought-resistant rootstocks improve scion performance through reduced oxidative stress, enhanced cellular defense, and transcriptional activation of stress-related genes. In the physiological thresholds and metabolic reprogramming section, *Medicago ruthenica* germplasm responses to saline–alkali stress is summarized, highlighting tolerant genotypes, key metabolites, higher germination vigor, and reduced oxidative damage. The okra panel depicts salinity-dependent hormetic growth, inhibition of germination at higher NaCl levels, and transcriptomic activation of ion homeostasis, calcium signaling, ROS detoxification, and cytoskeletal remodeling under mild stress. Together, these findings illustrate how multi-level regulatory networks coordinate stress adaptation and provide targets for breeding stress-resilient crops.

Collectively, these studies reveal proline metabolism, ion homeostasis (e.g., *HKT*/*SOS*/*NHX*), transcriptional regulation (e.g., *CPP*, *WRKY*, *NAC*), and metabolic reprogramming as conserved tolerance pillars across plant species, as illustrated in our integrated thematic model (**Figure 1**). Through the integration of GWAS, proteomics, and functional validation, these works provide breeders with precise molecular targets for enhancing underrepresented crops like safflower, kiwifruit, okra, and *Medicago*. Furthermore, grafting emerges as a robust and immediate agronomic solution that can be deployed alongside genetic gains to mitigate abiotic stress. While significant progress has been made, cross-species network conservation requires further validation, and combined salinity-drought studies remain scarce, necessitating longitudinal phenotyping to evaluate field performance under multi-stress conditions. Ultimately, the future integration of CRISPR editing of identified hub genes (such as *AcP5CS1* and *HKT1;5*) with AI-driven omics modeling will accelerate the translation to these molecular insights into high-yielding, climate-resilient cultivars.
